# Only connect: the merger of *BMC Pharmacology* and *BMC Clinical Pharmacology*

**DOI:** 10.1186/2050-6511-13-1

**Published:** 2012-08-13

**Authors:** Elizabeth C Moylan, Christopher Morrey, Joanne M Appleford-Cook

**Affiliations:** 1 BioMed Central Ltd, 236 Gray’s Inn Road, London, WC1X 8HL, UK

## Abstract

This editorial celebrates the launch of *BMC Pharmacology and Toxicology* within the *BMC* series of journals published by BioMed Central. The scope of the journal is interdisciplinary encompassing toxicology, experimental and clinical pharmacology including clinical trials. In this editorial we discuss the origins of this new journal and the ethos and policies under which it will operate.

## Aims and scope

This month, our two pharmacologically-based titles within the *BMC* series, *BMC Pharmacology* and *BMC Clinical Pharmacology,* join forces under the new title *BMC Pharmacology and Toxicology*. The scope of the combined journal is broad, retaining the full scopes of the original titles while also expanding to explicitly include the field of toxicology. The decision to merge the journals was taken in response to feedback received from key opinion leaders in the field and in consultation with our Editorial Boards. The vast majority were in favour of a wide-ranging journal, offering researchers the opportunity to submit to and browse a general journal with a wider audience than either of its component parts. A selection of the feedback we received is given below.

“I think the merger is an excellent idea, especially since the borders between pharmacology, clinical pharmacology and toxicology can be rather ‘blurry’ and because of clinical and translational sciences being a major effort in the US and elsewhere”

Paul Insel, University of California, San Diego, USA

“The policy of launching the new *BMC Pharmacology and Toxicology* journal is a far-sighted and modern view and certainly the adjourned scopes and the new sections are more appealing since they cover more adequately the inter-related fields of Pharmacology and Toxicology”

Giorgio Palu, Universtiy of Padua, Italy

“Launching the merged journal is an excellent opportunity to publish cutting-edge research on the border of pharmacology and toxicology, which is essential for modern drug discovery”

Maria Miteva, Inserm - University Paris Diderot, France

“This merger would allow different readers, in terms of cultural background and scientific interest, to get new knowledge and expand their vision of science”

Maurizio Memo, University of Brescia Medical School, Italy

The merger not only reflects the growing cross-disciplinary nature of the field in this era of translational medicine, but also to paraphrase Gregory Petsko, recognises that *“sometimes bigger is better, less is more, especially in an age where we need to think and read as broadly as we can”*[1]. We hope that the merger of *BMC Pharmacology* and *BMC Clinical Pharmacology* will facilitate connections across pre-clinical and clinical research and that the communities which supported the individual journals for the last 10 years will also support this new venture. We welcome submissions which span the fields of experimental and clinical pharmacology; toxicology; drug design, discovery and delivery, and computational, *in silico* and modelling studies.

*BMC Pharmacology and Toxicology* will maintain the ethos of its predecessors and the rest of the *BMC* series journals, and continue to publish work deemed by peer reviewers to be a coherent and sound addition to scientific knowledge. There will be less emphasis on interest levels, provided that the research constitutes a useful contribution to the field [2]. All previous content will remain open access and be freely accessible from the new journal platform.

As with other journals in the *BMC* series, *BMC Pharmacology and Toxicology* has an international Editorial Board which retains many of the previous members of *BMC Pharmacology* and *BMC Clinical Pharmacology* with additional new faces. The journal is divided into a number of subject-specific sections under the stewardship of academic Section Editors, who will be supported by academic Associate Editors and Editorial Advisors as well as in-house Editors. Dividing the journal into sections not only allows authors to submit to a specific section but allows readers to browse articles in a given section of interest to them and in their field [3]. As an online journal we also make use of article level metrics to indicate the most relevant and important publications to our readers [4] not least because research published open access has the potential to reach a much wider range of readers than a subscription-based journal [5]. Publishing electronically also makes full use of digital technologies and permits the inclusion of large datasets and links to animations and video clips.

## Openness

*BMC Pharmacology and Toxicology* will operate an open-peer review policy as is currently the case on all medical titles within the *BMC* series [6]. There are two levels to this “openness”. The first is that authors will see the reviewers' names; the second is that the reading public will also see who reviewed the article and how the authors responded, if the article is published. This will be available as part of the pre-publication history of the published article.

Research into the effect of open peer review suggests numerous benefits, the most important of which are accountability, fairness, and giving credit to reviewers for their efforts [7-9]. However, we recognise that there are negatives also. Some (junior) reviewers may feel uncomfortable signing a critical report, especially when recommending rejection [10]. The reluctance to open review also means that more potential peer reviewers have to be invited to review a manuscript openly than under a peer review system which is closed Parkin EC *et al.* unpublished observations, [10-12]. However, given the unique situation created by merging *BMC Pharmacology* (with closed peer review) and *BMC Clinical Pharmacology* (with open peer review) we have opted to make the peer review process open and we will report on our findings in the next two years. We believe that for a journal publishing research frequently sponsored by pharmaceutical companies the potential benefits to openness outweigh the negatives. By making peer review completely transparent, we aim to reduce the competing interests that can occur.

*BMC Pharmacology and Toxicology* also supports initatives to promote open data sharing [13,14]. Our publisher, BioMedCentral, offers an annual Open Data Award to recognize researchers who have published in BioMed Central journals and have demonstrated leadership in the sharing, standardization, publication, or re-use of biomedical research data [15]. This is particularly relevant given recent calls for policy reform of the US Food and Drug Administration with respect to data sharing on abandoned drugs which has the potential to make the search for effective drugs more efficient [16].

## Looking ahead

To launch *BMC Pharmacology and Toxicology*, we are publishing the first in an occasional series of special review articles. Our first review by Terry Kenakin focuses on the potential for selective pharmacological therapies based on the discovery that not all agonists uniformly activate signalling pathways resulting in biased signalling [17]. In the coming months, we will also be publishing a series of interviews with our Section Editors in which they discuss some of the topics of particular interest within their subject-specific sections. In the first of these, Phil Biggin gives his personal view on the key issues in computational, in silico and modelling studies [18]. While the focus of the journal is to publish original sound research, we also welcome commentaries and correspondence that explore the expanding boundaries of this field and suggest new opportunities for collaboration between each discipline. The launch of *BMC Pharmacology and Toxicology* marks the start of the next phase of growth for journal. We do hope you will take the time to visit the website and consider us for your future submissions.

## Competing interests

All authors are employees of BioMed Central.

## Authors’ contributions

All authors have been working on *BMC Pharmacology and Toxicology* and have contributed to this editorial. All authors read and approved the final text.

## Pre-publication history

The pre-publication history for this paper can be accessed here:

http://www.biomedcentral.com/2050-6511/13/1/prepub
